# Thirteen-Week Study of PM014 Subchronic Oral Toxicity in Rats

**DOI:** 10.1155/2014/189673

**Published:** 2014-07-01

**Authors:** Hwan-Suck Chung, Hyunil Lee, Hyunsu Bae

**Affiliations:** Department of Physiology, College of Korean Medicine, Kyung Hee University, No. 1 Hoeki-Dong, Dongdaemoon-gu, Seoul 130-701, Republic of Korea

## Abstract

PM014 is a modified herbal formula based on Chung-Sang-Bo-Ha-Tang, which is a well-known prescription drug used for curing various inflammatory pulmonary diseases. We previously showed that PM014 attenuated lung inflammation in a murine model of chronic obstructive pulmonary disease (COPD). The objective of the present study was to evaluate the chronic oral toxicity of PM014 in rats. PM014 was administered to rats orally once daily at doses of 0, 750, 1500, and 3000 mg/kg/day for 13 weeks. The PM014 treatment did not result in any toxicologically significant changes between the control and treatment groups in body weight, clinical signs, food consumption, dermatological and serum biochemical parameters, organ weight ratio, or histopathology. We concluded that no PM014-related toxicity was detected even at the highest doses investigated in this repeated dose oral toxicity study. Based on our results, the no-observed-adverse-effect level (NOAEL) of PM014 was 3000 mg/kg/day in both genders. These results might provide supportive evidence of the safety of PM014 to develop a new drug.

## 1. Introduction

PM014 is a drug modified from Chung-Sang-Bo-Ha-Tang (CSBHT). CSBHT has been used to treat chronic pulmonary diseases in Korea for centuries [1]. Previously, we developed the formulation of PM014 based on a series of* in vitro* and* in vivo* screening efforts.

PM014 contains seven species of medicinal plants (Table 1). We combined seven species of herbal extract which were produced by Sun Ten Pharmaceutical Co. and named them PM014. We previously conducted DART-MS (direct analysis in real time-mass spectrometry) on the PM104 preparation to reconfirm the composition of the herbal medicines [2]. Among the seven components of PM014, Moutan cortex is not allowed for use as a food ingredient. There has been no report on the toxicity of Moutan cortex. Some reports showed that Moutan cortex has a protective effect on acute liver injury [3] and on acetaminophen-induced hepatotoxicity [4]. And there are many reports about the beneficial effects of paeonol, a major component of Moutan cortex, such as an anti-inflammatory effect [5], protection of nephrotoxicity by cisplatin [6], and a neuroprotective effect [7]. Only one of the seven components of PM014 has been analyzed for its toxicity. Jin et al. demonstrated that Armeniacae semen (AS), one of the components of PM014, had no mutagenicity* in vitro* or* in vivo* [8]. It has been known that AS has toxicity because it contains hydrogen cyanide and amygdalin, especially in its endocarp. Prebrewed AS has been traditionally used to remove endocarp. The amygdalin of prebrewed AS contained 10% of amygdalin compared to the extract that contained the endocarps. 50% lethal dose and approximate lethal dose of prebrewed AS extracts in female and male rats were 9279.50 mg/kg and 1,000~2,000 mg/kg [9]. Even though stemona alkaloids from Stemonae Radix have high insect toxicity [10], there is no report about mammalian toxicity. Besides the above mentioned 3 components of PM014, there is no toxicological study about the rest of the components of PM014. However, there are various medicinal effects about their major components, such as renal protective effect of shizandrin [11] and hepatoprotective effect of baicalin [12]. The determination of the safety of PM014, which is a necessary step in the progression of this herbal medicine for use as a new drug, has not yet occurred. The aim of this study was to investigate the toxicity of PM014 by repeatedly administering it orally to rats for 13 weeks. In our previous single-dose oral toxicity study of PM014, the approximate lethal dose of PM014 was determined to be higher than 5000 mg/kg. In the 4-week repeated oral toxicity study at doses of 800, 2000, and 5000 mg/kg/day, the decrease of Na^+^ in the female 5000 mg/kg/day group was considered as PM014-related change with dose-response relationship (unpublished data). Although the statistical significance was not confirmed, the increasing tendency of the liver weight was observed in the 2000 and 5000 mg/kg/day groups. Based on these results, the high dose in this study was set at 3000 mg/kg/day which was expected to induce the liver toxicity. We hoped to find the no-observed-adverse-effect level (NOAEL), the toxic dose (TD), the maximum tolerated dose (MTD), and the target organs of the drug.

## 2. Materials and Methods

### 2.1. Animals

In the 13-week oral toxicity study, 48 male and 48 female Sprague-Dawley (SD) five-week-old specific pathogen-free (SPF) rats were obtained from Koatec Inc (Gyeonggi-do, Korea). Environmental controls were set to maintain the following conditions: temperature range of 23 ± 3°C, relative humidity range of 55 ± 15%, ventilation of 10–20 air changes/hr, 150–300 Lux of luminous intensity, and a 12 hr light/12 hr dark cycle.

### 2.2. Experimental Design

The 13-week oral dose study in rats was performed to assess the general toxicity of PM014 in rats (*n* = 10/sex/dose group) at doses of 0, 750, 1500, and 3000 mg/kg following daily administration by gavage. The animals were randomized by sex into one of the four dosage groups. The negative control group received saline in a volume of 12 mL/kg body weight (b.w.) by gavage. The PM014 was dissolved in saline and administered to the SD rats via oral gavage. After PM014 was administered orally at dosages of 0, 750, 1500, and 3000 mg/kg/day to 10 rats per group (male 10, female 10) for 13 weeks, several parameters, such as mortality, clinical signs, body weight changes, food and water consumption, urinalysis, hematology and serum biochemistry, necropsy findings, and relative organ weights, were observed. This study was conducted in accordance with two standards: The Korea Food and Drug Administration (KFDA) Notification no. 2005-60 “The Standards of Toxicity Study for Medicinal Products” (October 21, 2005) and The KFDA Notification no. 2005-79 “Good Laboratory Practice (GLP)” (December 21, 2005).

### 2.3. Clinical Signs

Each animal was observed at least once a day for clinical signs and mortalities, and the observations were recorded individually.

### 2.4. Body Weight and Food Consumption

Animals were weighed on day 1 before the administration and from then once a week and on the day of necropsy. The body weight for the necropsy was measured after fasting overnight. The food consumption was measured on day 1 and once a week during the in-life phase. Rats were given a known quantity of food by cage, and the difference was measured the next day to calculate the mean daily consumption (g/head/day).

### 2.5. Blood Analysis

Blood samples for hematology and clinical biochemistry were collected from the posterior vena cava of all animals under deep isoflurane anesthesia (Ifran liquid, Hana Pharm. Co., Ltd) on the day of necropsy. Animals were fasted overnight (with water available) prior to the sample collection. For hematological test, approximately 0.3 mL whole blood was placed into a CBC bottle (Vacutainer 3 mL, BD, USA) that contained anticoagulant EDTA-2K. Then, the following parameters were detected with a coulter counter (ADVIA 2120, Siemens, USA). The following parameters were measured in seconds from the plasma by the nephelometric analysis method with a coagulation time analyzer (ACL 100, Instrumentation Laboratory, USA): red blood cell (RBC), platelet count (PLT), hematocrit (HCT), white blood cell (WBC), red cell distribution width (RDW), Hb concentration distribution width (HDW), hemoglobin concentration (HGB), neutrophil (NEU), mean corpuscular volume (MCV), lymphocyte (LYM), mean cell Hemoglobin (MCH), monocyte (MONO), mean cell hemoglobin concentration (MCHC), eosinophil (EOS), mean platelet volume (MPV), basophil (BASO), and large unstained cells (LUC).

For the clinical biochemistry test, a portion of the blood was added into a 5 mL Vacutainer tube (Insepack, Sekisui, Japan) that contained a clot activator. The blood was coagulated by remaining at room temperature for 15–20 min and then centrifuged (3,000 RPM, 1,630 RCF, MF300, Hanil, Korea) for 10 min. The following parameters were measured: aspartate aminotransferase (AST), albumin (ALB), alanine aminotransferase (ALT), albumin/globulin ratio (A/G ratio), alkaline phosphatase (ALP), blood urea nitrogen (BUN), creatine phosphokinase (CPK), creatinine (CRE), total bilirubin (TBIL), inorganic phosphorus (IP), glucose (GLU), calcium (Ca^2+^), total cholesterol (TCHO), sodium (Na^+^), triglyceride (TG), potassium (K^+^), total protein (TP), and chloride (Cl^−^).

### 2.6. Urinalysis

Before the initiation of drug administration and two days before the necropsy, each rat was placed in a metabolic cage for 3 h to collect urine. Urine was examined using a urinalysis test strip (Bayer) and an automatic urinalysis system (CliniTek 100, Bayer). The urinalysis test items were glucose, bilirubin, ketone bodies, specific gravity, occult blood, pH, proteins, urobilinogen, nitrites, and leukocytes.

### 2.7. Autopsy and Histopathological Study

The animals were sacrificed by exsanguination and dissected. During the dissection, the color, texture, and lump of the parenchymatous organs were carefully examined. The color and integrity of the mucosa of the body cavities were also examined. Additionally, the weights of the brain, hypothyroid glands, lungs, heart, thymus glands, liver, spleen, kidneys, adrenal glands, prostate, testicle, and ovaries were measured and recorded. The organ-body index was calculated according to the following formula [13]: organ-body index (%) = wet organ weight/body weight × 100%.

All of the extracted organs from all of the animals were fixed in a 10% neutral formaldehyde solution, with the following exception: the testes were fixed in Bouin's solution.

### 2.8. Statistical Analysis

To compare the test groups with the control group, parametric and nonparametric multiple comparison procedures were applied. All statistical analyses were performed with the widely used commercial statistical package SPSS 10.1. In the case of continuous data, such as body weight, food and water consumption, hematology and serum biochemistry, and relative organ weight, the data were expressed as the means ± SD and were subjected to the one-way ANOVA to determine significance. When a significant difference was found, it was subjected to the Levene test for equality of variances. If homoscedasticity of the data was accepted, Duncan's multiple range test was applied. Otherwise, Dunnett's *t*-test was conducted to examine the significance of differences between the experimental and control groups.

## 3. Results

**Figure 1:**
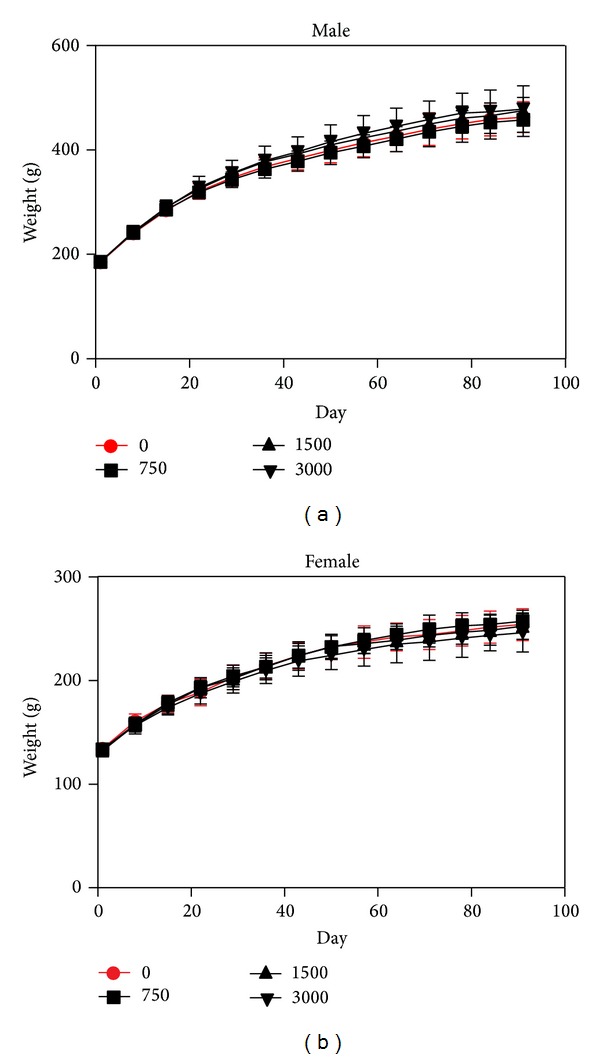
Growth curve of male and female rats administered PM014. Body weight of the PM014 treated rats was recorded at every week for 13 weeks. The data shown are the means ± SD (*n* = 10/group).

**Figure 2:**
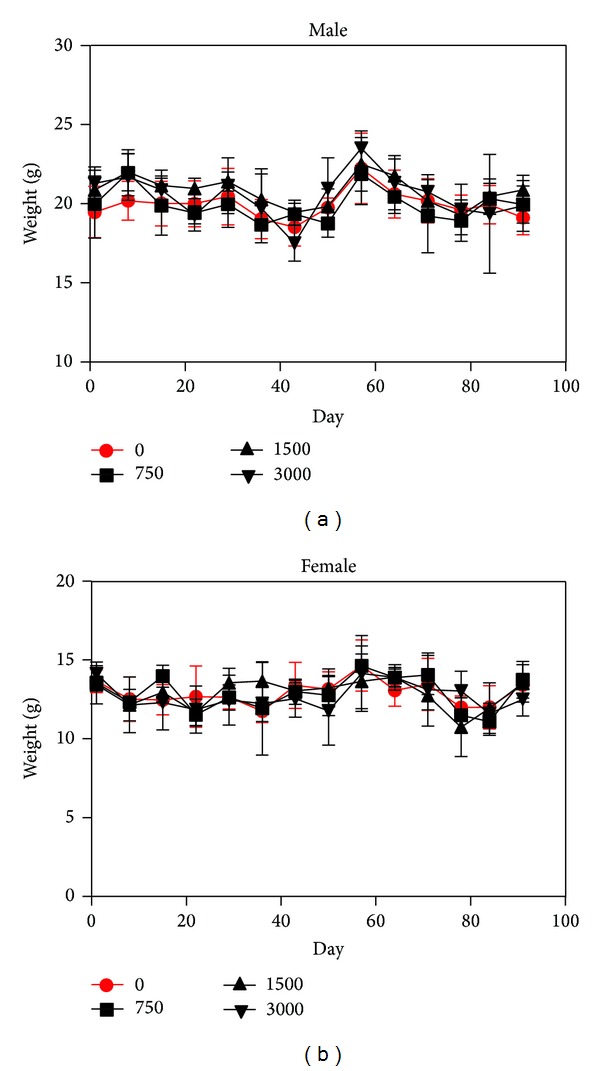
Food consumption of the rats over the 13-week PM014 administration. Food consumption of the PM014 treated rats was recorded at every week for 13 weeks. The data shown are the means ± SD (*n* = 10/group).

There was no mortality or clinical changes related to the 13-week administration of PM014. No significant difference in body weight or food consumption was found between the animals treated with PM014 and the controls (Figures 1 and 2).

The hematological examination (Table 2) showed that the WBC values were significantly increased in both the male and female rats of the 750 and 3000 mg/kg/day PM014 treatment groups; however, there was no inflammatory response or increase in hematogenesis related to the observed increase in WBC values upon histopathological exam. Because the WBC values were within the normal range, these changes were not considered to be related to the toxicity of PM014 administration. The hematocrit (HCT) and mean corpuscular hemoglobin (MCH) values in the female rats of the 750 mg/kg/day PM014 treatment group were significantly lower than those of the control group. The distribution width (HDW) and hemoglobin concentration (HGB) of the female rats of the 3000 mg/kg/day PM014 treatment group were significantly lower than those of the control group but were within the normal range. Because a change in the RBC level, which is considered to be related to changes in HDW and HGB, was not observed in these groups, those changes appeared to be unrelated to the administration of PM014.

The chemical analysis of the blood showed that the alkaline phosphatase (ALP) and total bilirubin (TBIL) levels were significantly increased in the female rats of the 3000 mg/kg/day PM014 treatment group (Table 3); however, those values were within the normal range and there was no change in the liver on histopathological exam, indicating that those changes were unrelated to the administration of PM014.

Although the relative weight of the liver of the male rats in the 3000 mg/kg/day PM014 treatment group was significantly higher than that of the control group (Table 4), it was within normal range; there was no consistent difference in the female rats of that group and no morphological change on histopathological exam was observed. Thus, this change in weight was considered to be unrelated to the PM014 administration.

## 4. Discussion

Toxicity studies are required when developing a new herbal drug. This study aimed at examining the subchronic toxicity profile of PM014 in rats. After PM014 was administered orally at dosages of 750, 1500, and 3000 mg/kg/day to 10 animals per group (ten males, ten females) for 13 weeks, several parameters such as mortality, clinical signs, body weight changes, food and water consumption, urinalysis, hematology and serum biochemistry, necropsy findings, and relative organ weights were observed. To determine whether the findings were normal, these data were compared with reference data from a study performed at the preclinical research center ChemOn Inc. between 2005 and 2008.

The hematological results showed significant WBC changes in the male rats in the 750 and 3000 mg/kg/day PM014 dosage groups (10.06, 10.07, resp.). The hematocrit (HCT) and mean corpuscular hemoglobin (MCH) values in the females of the 750 mg/kg/day PM014 dosage group were significantly lower than those of the control group (41.8 and 18.3 lower than control, resp.). The Hb concentration distribution width (HDW) and hemoglobin concentration (HGB) values in the females of the 3000 mg/kg/day PM014 dosage group were significantly lower than the control group (2.33 and 14.0 lower than control, resp.). Because these values were within the normal range (WBC: 4.8~20.1; HCT: 36.9~51.8; MCH: 16.7~21.8; HDW: 1.8~2.7; HGB1: 3.1~17.0) and there were no histopathological changes related to them, such as an inflammatory response or hematogenesis [14, 15], the changes were considered unrelated to the PM014 administration.

The blood chemical examination showed a significant increase in the ALP (70.9 U/L) and TBIL (0.23 mg/dL) levels in the females of the 3000 mg/kg/day PM014 treatment group; however, these values were within the normal range (ALP: 30.1~116.0 U/L; TBIL: 0.15~0.35 mg/dL) [15], and there were no histopathological changes to the liver. Thus, those changes appeared to be unrelated to the administration of PM014.

Although the relative weight of the livers in the males of the 3000 mg/kg/day PM014 treatment group was significantly higher than that of the control group (2.8191%), this difference did not exist when the data were matched for sex, the values were within the normal range (2.1591~2.9796%) [15], and no morphological change was observed during the histopathological exam. Thus, this change was considered to be unrelated to PM014 administration.

Because the effective dose range of PM014 was 50–200 mg/kg based on our previous pharmacological study [16] and there was no toxicity up to 3000 mg/kg/day of PM014, we expect that PM014 would be safe without any adverse effects within the effective dose range.

In conclusion, the repeated oral administration to rats of PM014 at dosages up to 3000 mg/kg/day for 13 weeks resulted in no toxicological changes in any of the examinations, namely, mortality, clinical signs, body weight changes, food and water consumption, urinalysis, hematology and serum biochemistry examinations, necropsy findings, and relative organ weights. There was no systemic or toxicologically significant change related to PM014 administration in the present 13-week repeated dose study and the NOAEL (no-observed-adverse-effect level) was 3000 mg/kg/day.

## Figures and Tables

**Table 1:** Components of PM014.

Herb	Pharmaceutical name	Amount (g)
Suckjihwag	Rehmannia Radix Preparata	600
Mockdanpi	Moutan cortex	300
Omija	Schisandrae fructus	300
Chunmundong	Asparagi Tuber	300
Hengin	Armeniacae semen	225
Hwangkum	Scutellariae Radix	225
Baekbukuen	Stemonae Radix	150
Total		**2,100 g**

**Table 2:** Hematological parameters of rats treated orally with PM014 for 13 weeks.

Hematological parameters	PM014 (mg/kg/day)			
	0	750	1500	3000
Males				
RBC (10^6^/*μ*L)	8.98 ± 0.33	9.25 ± 0.55	8.93 ± 0.24	8.93 ± 0.29
HGB (g/dL)	15.7 ± 0.6	15.5 ± 0.9	15.3 ± 0.4	15.4 ± 0.5
HCT (%)	47.6 ± 1.9	47.1 ± 2.5	46.3 ± 1.3	46.9 ± 1.6
MCV (fL)	53.0 ± 1.7	51.1 ± 3.6	51.9 ± 1.5	52.6 ± 1.6
MCH (pg)	17.5 ± 0.5	16.8 ± 1.4	17.1 ± 0.5	17.2 ± 0.6
MCHC (g/dL)	33.0 ± 0.3	32.9 ± 0.6	32.9 ± 0.4	32.8 ± 0.4
RDW (%)	12.4 ± 0.3	12.8 ± 0.4	12.6 ± 0.4	12.3 ± 0.3
HDW (g/dL)	2.50 ± 0.11	2.57 ± 0.09	2.57 ± 0.12	2.54 ± 0.20
RET (%)	2.34 ± 0.28	2.37 ± 0.32	2.48 ± 0.41	2.59 ± 0.31
PLT (10^3^/*μ*L)	934.9 ± 60.3	1018.8 ± 60.5	978.3 ± 92.7	1001.9 ± 102.2
MPV (fL)	4.7 ± 0.1	4.7 ± 0.2	4.7 ± 0.1	4.8 ± 0.2
WBC (10^3^/*μ*L)	7.98 ± 1.40	10.06 ± 1.72**	9.09 ± 1.74	10.07 ± 1.42**
NEU (%)	22.5 ± 4.9	22.9 ± 4.5	20.8 ± 4.4	19.8 ± 7.6
LYM (%)	70.6 ± 5.3	70.1 ± 5.3	72.5 ± 5.0	73.9 ± 7.3
MONO (%)	4.72 ± 1.21	4.95 ± 1.24	4.90 ± 1.34	4.62 ± 0.78
EOS (%)	1.60 ± 0.49	1.51 ± 0.45	1.17 ± 0.49	1.15 ± 0.49
BASO (%)	0.16 ± 0.05	0.17 ± 0.05	0.19 ± 0.03	0.19 ± 0.06
LUC (%)	0.34 ± 0.16	0.37 ± 0.12	0.37 ± 0.12	0.40 ± 0.24
PT (sec)	8.90 ± 0.28	8.87 ± 0.18	8.82 ± 0.24	8.87 ± 0.23
APTT (sec)	18.3 ± 0.6	18.3 ± 0.7	18.0 ± 0.9	18.1 ± 0.6
Females				
RBC (10^6^/*μ*L)	7.88 ± 0.17	7.77 ± 0.22	7.79 ± 0.22	7.62 ± 0.36
HGB (g/dL)	14.8 ± 0.3	14.2 ± 0.3	14.4 ± 0.4	14.0 ± 0.7*
HCT (%)	43.5 ± 1.1	41.8 ± 0.9**	42.5 ± 0.8	41.6 ± 2.0
MCV (fL)	55.2 ± 0.8	53.8 ± 1.3	54.6 ± 1.1	54.6 ± 1.4
MCH (pg)	18.8 ± 0.3	18.3 ± 0.3*	18.5 ± 0.5	18.4 ± 0.3
MCHC (g/dL)	34.0 ± 0.3	33.9 ± 0.5	33.8 ± 0.7	33.8 ± 0.7
RDW (%)	11.4 ± 0.2	11.0 ± 0.3	11.2 ± 0.3	11.1 ± 0.4
HDW (g/dL)	2.51 ± 0.12	2.34 ± 0.14	2.35 ± 0.17	2.33 ± 0.10*
RET (%)	3.16 ± 0.45	2.56 ± 0.48	2.87 ± 0.49	2.98 ± 0.96
PLT (10^3^/*μ*L)	1105.3 ± 95.7	1137.4 ± 113.6	1128.8 ± 154.8	1094.3 ± 112.5
MPV (fL)	4.7 ± 0.1	4.9 ± 0.2	5.0 ± 0.4	4.9 ± 0.2
WBC (10^3^/*μ*L)	4.93 ± 1.01	5.39 ± 2.03	5.26 ± 0.54	4.90 ± 1.03
NEU (%)	12.4 ± 4.0	18.2 ± 8.4	14.9 ± 2.8	15.8 ± 6.3
LYM (%)	80.5 ± 4.9	74.9 ± 9.0	77.0 ± 3.8	77.8 ± 6.5
MONO (%)	4.80 ± 1.23	4.91 ± 1.02	5.88 ± 1.35	4.34 ± 0.93
EOS (%)	1.72 ± 0.66	1.45 ± 0.34	1.72 ± 0.35	1.52 ± 0.62
BASO (%)	0.13 ± 0.07	0.08 ± 0.06	0.12 ± 0.09	0.11 ± 0.06
LUC (%)	0.37 ± 0.11	0.40 ± 0.13	0.47 ± 0.13	0.46 ± 0.08
PT (sec)	9.00 ± 0.28	9.06 ± 0.27	8.99 ± 0.27	9.15 ± 0.26
APTT (sec)	17.9 ± 0.7	17.9 ± 1.1	18.5 ± 0.7	17.9 ± 0.5

RBC: red blood cell; HGB: haemoglobin concentration; HCT: hematocrit; MCV: mean corpuscular volume; MCH: mean corpuscular hemoglobin; MCHC: mean corpuscular hemoglobin concentration; RDW: red cell distribution width; HDW: Hb concentration distribution width; RET: reticulocytes; PLT: platelet; MPV: mean platelet volume; WBC: white blood cells; NEU: neutrophil; LYM: lymphocyte; MONO: monocyte; EOS: eosinophil; BASO: basophil; LUC: large unstained cells; PT: prothrombin time; APTT: activated partial thromboplastin time. The data shown are the means ± SD (*n* = 10/group). *P < 0.05, **P < 0.01 compared with the control group.

**Table 3:** Biochemical parameters of rats treated orally with PM014 for 13 weeks.

Biochemical parameters	PM014 (mg/kg/day)			
	0	750	1500	3000
Males				
AST (U/L)	86.3 ± 7.7	85.9 ± 12.3	85.5 ± 6.8	87.5 ± 12.8
ALT (U/L)	40.5 ± 4.3	40.1 ± 4.5	39.8 ± 4.9	42.3 ± 9.0
ALP (U/L)	74.4 ± 18.4	78.7 ± 10.3	78.9 ± 15.5	81.4 ± 12.2
CPK (U/L)	130.5 ± 62.4	118.1 ± 69.7	120.6 ± 65.1	109.9 ± 62.0
TBIL (mg/dL)	0.17 ± 0.02	0.19 ± 0.03	0.20 ± 0.02	0.19 ± 0.01
GLU (mg/dL)	156.1 ± 17.0	145.3 ± 13.7	151.4 ± 24.9	145.2 ± 18.7
TCHO (mg/dL)	112.2 ± 17.5	113.0 ± 25.3	106.2 ± 12.3	111.0 ± 23.4
TG (mg/dL)	40.7 ± 11.3	41.3 ± 9.2	43.3 ± 11.8	46.6 ± 12.0
TP (g/dL)	6.48 ± 0.36	6.51 ± 0.33	6.44 ± 0.11	6.32 ± 0.18
ALB (g/dL)	3.11 ± 0.17	3.10 ± 0.08	3.08 ± 0.06	3.02 ± 0.11
A/G (ratio)	0.92 ± 0.03	0.91 ± 0.07	0.92 ± 0.03	0.91 ± 0.03
BUN (mg/dL)	18.6 ± 2.5	17.5 ± 2.3	18.6 ± 2.2	18.6 ± 3.1
CRE (mg/dL)	0.50 ± 0.04	0.49 ± 0.04	0.50 ± 0.04	0.47 ± 0.02
IP (mg/dL)	6.66 ± 0.54	6.71 ± 0.43	6.80 ± 0.62	6.79 ± 0.42
Ca^2+^ (mg/dL)	10.55 ± 0.38	10.79 ± 0.37	10.61 ± 0.26	10.54 ± 0.18
Na^+^ (mmol/L)	139.71 ± 1.54	139.74 ± 1.14	139.94 ± 0.98	139.69 ± 1.00
K^+^ (mmol/L)	4.90 ± 0.60	4.72 ± 0.31	4.85 ± 0.51	4.74 ± 0.27
Cl^−^ (mmol/L)	101.88 ± 1.51	101.18 ± 1.20	101.88 ± 0.98	102.32 ± 1.19
Females				
AST (U/L)	89.5 ± 10.9	86.1 ± 9.8	91.0 ± 23.2	88.7 ± 10.3
ALT (U/L)	36.4 ± 5.2	34.9 ± 5.0	40.1 ± 10.5	36.6 ± 5.2
ALP (U/L)	55.0 ± 14.3	63.8 ± 11.8	61.1 ± 8.5	70.9 ± 10.6**
CPK (U/L)	126.8 ± 47.3	123.7 ± 71.7	104.0 ± 33.2	122.5 ± 58.0
TBIL (mg/dL)	0.20 ± 0.02	0.21 ± 0.03	0.21 ± 0.02	0.23 ± 0.03*
GLU (mg/dL)	123.1 ± 13.0	125.1 ± 13.9	120.4 ± 11.2	115.5 ± 5.5
TCHO (mg/dL)	114.6 ± 24.8	108.1 ± 18.2	98.2 ± 9.2	96.6 ± 15.3
TG (mg/dL)	41.7 ± 11.0	33.7 ± 4.6	35.5 ± 7.8	33.5 ± 6.2
TP (g/dL)	6.37 ± 0.35	6.31 ± 0.31	6.27 ± 0.17	6.24 ± 0.29
ALB (g/dL)	3.30 ± 0.21	3.27 ± 0.22	3.29 ± 0.11	3.26 ± 0.15
A/G (ratio)	1.08 ± 0.04	1.08 ± 0.07	1.10 ± 0.06	1.10 ± 0.05
BUN (mg/dL)	21.1 ± 3.4	19.2 ± 1.8	20.1 ± 2.5	20.2 ± 3.1
CRE (mg/dL)	0.60 ± 0.06	0.62 ± 0.05	0.60 ± 0.06	0.59 ± 0.06
IP (mg/dL)	5.50 ± 0.66	5.68 ± 0.72	6.24 ± 1.16	6.19 ± 0.49
Ca^2+^ (mg/dL)	10.10 ± 0.28	10.09 ± 0.23	10.21 ± 0.20	10.05 ± 0.24
Na^+^ (mmol/L)	139.53 ± 1.43	139.17 ± 0.94	139.81 ± 1.51	139.43 ± 1.09
K^+^ (mmol/L)	4.15 ± 0.19	4.18 ± 0.37	4.38 ± 0.51	4.40 ± 0.34
Cl^−^ (mmol/L)	103.89 ± 1.47	103.94 ± 1.34	104.60 ± 1.06	104.79 ± 1.38

AST: aspartate aminotransferase; ALT: alanine aminotransferase; ALP: alkaline phosphatase; CPK: creatine phosphokinase; TBIL: total bilirubin; GLU: glucose; TCHO: total cholesterol; TG: triglyceride; TP: total protein; ALB: albumin; A/G: albumin/globulin ratio; BUN: blood urea nitrogen; CRE: creatinine; IP: inorganic phosphorus. The data shown are the means ± SD (*n* = 10/group).

*P < 0.05, **P < 0.01 compared with the control group.

**Table 4:** Relative organ weights of rats treated orally with PM014 for 13 weeks.

Relative organ weights (%)	PM014 (mg/kg/day)			
	0	750	1500	3000
Males				
Adrenal gland, left	0.0058 ± 0.0006	0.0063 ± 0.0007	0.0059 ± 0.0009	0.0062 ± 0.0011
Adrenal gland, right	0.0056 ± 0.0006	0.0061 ± 0.0006	0.0057 ± 0.0007	0.0060 ± 0.0010
Pituitary gland	0.0027 ± 0.0003	0.0029 ± 0.0003	0.0029 ± 0.0003	0.0029 ± 0.0003
Thymus	0.0712 ± 0.0151	0.0767 ± 0.0191	0.0703 ± 0.0093	0.0731 ± 0.0215
Prostate gland	0.1347 ± 0.0358	0.1664 ± 0.0347	0.1273 ± 0.0369	0.1532 ± 0.0238
Testis, left	0.4398 ± 0.1099	0.4890 ± 0.0398	0.4665 ± 0.0373	0.4437 ± 0.0266
Testis, right	0.4731 ± 0.0310	0.4836 ± 0.0309	0.4644 ± 0.0316	0.4561 ± 0.0275
Epididymis, left	0.1500 ± 0.0300	0.1667 ± 0.0163	0.1490 ± 0.0140	0.1474 ± 0.0119
Epididymis, right	0.1560 ± 0.0092	0.1661 ± 0.0107	0.1487 ± 0.0121	0.1530 ± 0.0126
Spleen	0.1858 ± 0.0158	0.1923 ± 0.0260	0.2047 ± 0.0304	0.2165 ± 0.0234
Kidney, left	0.3012 ± 0.0170	0.3182 ± 0.0233	0.2956 ± 0.0143	0.3064 ± 0.0174
Kidney, right	0.3119 ± 0.0219	0.3288 ± 0.0282	0.3064 ± 0.0151	0.3151 ± 0.0123
Heart	0.3247 ± 0.0304	0.3308 ± 0.0313	0.3267 ± 0.0254	0.3348 ± 0.0360
Lung	0.4083 ± 0.0226	0.4148 ± 0.0426	0.4091 ± 0.0359	0.3977 ± 0.0290
Brain	0.4404 ± 0.0196	0.4456 ± 0.0338	0.4381 ± 0.0256	0.4316 ± 0.0413
Liver	2.5858 ± 0.1432	2.6340 ± 0.1517	2.6295 ± 0.1530	2.8191 ± 0.1575**
Females				
Ovary, left	0.0177 ± 0.0024	0.0179 ± 0.0026	0.0181 ± 0.0019	0.0158 ± 0.0026
Ovary, right	0.0190 ± 0.0029	0.0191 ± 0.0028	0.0189 ± 0.0019	0.0178 ± 0.0021
Adrenal gland, left	0.0120 ± 0.0019	0.0131 ± 0.0019	0.0128 ± 0.0016	0.0121 ± 0.0015
Adrenal gland, right	0.0116 ± 0.0010	0.0129 ± 0.0019	0.0121 ± 0.0016	0.0115 ± 0.0013
Pituitary gland	0.0054 ± 0.0003	0.0059 ± 0.0007	0.0056 ± 0.0004	0.0056 ± 0.0006
Thymus	0.1142 ± 0.0186	0.1012 ± 0.0171	0.0977 ± 0.0215	0.1014 ± 0.0241
Uterus	0.3232 ± 0.1417	0.2807 ± 0.0870	0.3351 ± 0.1752	0.2860 ± 0.1053
Spleen	0.2424 ± 0.0174	0.2426 ± 0.0234	0.2447 ± 0.0434	0.2348 ± 0.0290
Kidney, left	0.2931 ± 0.0160	0.2977 ± 0.0139	0.2951 ± 0.0185	0.3005 ± 0.0149
Kidney, right	0.3087 ± 0.0079	0.3090 ± 0.0157	0.3381 ± 0.1099	0.3163 ± 0.0139
Heart	0.3746 ± 0.0292	0.3758 ± 0.0168	0.3837 ± 0.0159	0.3788 ± 0.0253
Lung	0.5589 ± 0.0299	0.5108 ± 0.1637	0.5573 ± 0.0435	0.5550 ± 0.0334
Brain	0.7315 ± 0.0587	0.7327 ± 0.0283	0.7397 ± 0.0518	0.7559 ± 0.0447
Liver	2.4427 ± 0.2687	2.5962 ± 0.1578	2.4736 ± 0.1612	2.4241 ± 0.1368

Relative organ weight was calculated using the following formula: organ-body index (%) = wet organ weight/body weight × 100%. The data shown are the means ± SD (*n* = 10/group).

**P < 0.01 compared with the control group.

## References

[1] Roh GS, Seo SW, Yeo S (2005). Efficacy of a traditional Korean medicine, Chung-Sang-Bo-Ha-Tang, in a murine model of chronic asthma. *International Immunopharmacology*.

[2] Lee H, Kim Y, Kim HJ (2012). Herbal formula, PM014, attenuates lung inflammation in a murine model of chronic obstructive pulmonary disease. *Evidence-Based Complementary and Alternative Medicine*.

[3] Park J, Kim H, Lee S (2011). Protective effects of Moutan Cortex Radicis against acute hepatotoxicity. *African Journal of Traditional, Complementary, and Alternative Medicines*.

[4] Shon Y, Nam KS (2004). Protective effect of Moutan Cortex extract on acetaminophen-induced hepatotoxicity in mice. *Journal of Ethnopharmacology*.

[5] Fu PK, Wu CL, Tsai TH, Hsieh CL (2012). Anti-inflammatory and anticoagulative effects of paeonol on LPS-induced acute lung injury in rats. *Evidence-Based Complementary and Alternative Medicine*.

[6] Lee H, Lee G, Kim H, Bae H (2013). Paeonol, a major compound of moutan cortex, attenuates Cisplatin-induced nephrotoxicity in mice. *Evidence Based Complementary and Alternative Medicine*.

[7] Liu J, Feng L, Ma D (2013). Neuroprotective effect of paeonol on cognition deficits of diabetic encephalopathy in streptozotocin-induced diabetic rat. *Neuroscience Letters*.

[8] Jin J, Liu B, Zhang H, Tian X, Cai Y, Gao P (2009). Mutagenicity of Chinese traditional medicine Semen Armeniacae amarum by two modified Ames tests. *BMC Complementary and Alternative Medicine*.

[9] Park JH, Seo BI, Cho SY (2013). Single oral dose toxicity study of prebrewed armeniacae semen in rats. *Toxicological Research*.

[10] Kaltenegger E, Brem B, Mereiter K (2003). Insecticidal pyrido[1,2-*a*]azepine alkaloids and related derivatives from *Stemona* species. *Phytochemistry*.

[11] Bunel V, Antoine MH, Nortier J (2013). Protective effects of schizandrin and schizandrin B towards cisplatin nephrotoxicity in vitro. *Journal of Applied and Toxicology*.

[12] Jang SI, Kim HJ, Hwang KM (2003). Hepatoprotective effect of baicalin , a major flavone from *Scutellaria radix*, on acetaminophen-induced liver injury in mice. *Immunopharmacology and Immunotoxicology*.

[13] Liu Z, Li C, Li M, Li D, Liu K (2004). The subchronic toxicity of hydroxysafflor yellow A of 90 days repeatedly intraperitoneal injections in rats. *Toxicology*.

[14] Petterino C, Argentino-Storino A (2006). Clinical chemistry and haematology historical data in control Sprague-Dawley rats from pre-clinical toxicity studies. *Experimental and Toxicologic Pathology*.

[15] Han XH, Kim ZZ, Ahn KH (2010). Reference data of the main physiological parameters in control sprague-dawley rats from pre-clinical toxicity studies. *Laboratory Animal Research*.

[16] Jung KH, Choi HL, Park S (2014). The effects of the standardized herbal formula PM014 on pulmonary inflammation and airway responsiveness in a murine model of cockroach allergen-induced asthma. *Journal of Ethnopharmacology*.

